# Nitric Oxide Signaling in Cardiovascular Physiology and Pathology: Mechanisms, Dysregulation, and Therapeutic Frontiers

**DOI:** 10.3390/ijms27020629

**Published:** 2026-01-08

**Authors:** Sakthipriyan Venkatesan, Carlo Smirne, Carmen Imma Aquino, Daniela Surico, Valentino Remorgida, Mohammad Mostafa Ola Pour, Mario Pirisi, Elena Grossini

**Affiliations:** 1 Laboratory of Physiology, Department of Translational Medicine, Università del Piemonte Orientale, Via Solaroli 17, 28100 Novara, Italy; sakthipriyan.venkatesan@uniupo.it (S.V.); 20046522@studenti.uniupo.it (M.M.O.P.); 2 Internal Medicine Unit, Department of Translational Medicine, Università del Piemonte Orientale, Via Solaroli 17, 28100 Novara, Italy; carlo.smirne@med.uniupo.it (C.S.); mario.pirisi@med.uniupo.it (M.P.); 3 Gynecology and Obstetrics Unit, Department of Translational Medicine, Università del Piemonte Orientale, 28100 Novara, Italy; c.immaquino@gmail.com (C.I.A.); daniela.surico@med.uniupo.it (D.S.); valentino.remorgida@med.uniupo.it (V.R.)

**Keywords:** cardiovascular disease, endothelial dysfunction, nitric oxide equilibrium, oxidative stress, therapeutics

## Abstract

Nitric oxide (NO), a fundamental gaseous signaling molecule, is indispensable for cardiovascular homeostasis. This review synthesizes the expansive field of NO biology within the unifying framework of Nitric Oxide Equilibrium (NOE), i.e., the critical balance between its synthesis, bioavailability, and degradation. In a physiological state, NOE maintains vascular health by regulating blood pressure, preventing thrombosis, suppressing inflammation, and optimizing both cardiac and mitochondrial function. Here, we analyze how NOE disruption, primarily through oxidative stress and enzymatic dysfunction, underlies the pathogenesis of major cardiovascular diseases, including atherosclerosis, heart failure, ischemia–reperfusion injury, and cerebrovascular diseases like stroke. A critical evaluation of therapeutic strategies designed to restore NOE is presented, encompassing classic NO donors and phosphodiesterase-5 inhibitors, alongside next-generation soluble guanylate cyclase modulators and precision nanomedicine approaches. By identifying key knowledge gaps and methodological hurdles, this review charts a course for future research focused on biomarker-guided interventions and personalized medicine. Ultimately, we frame the restoration of NOE as a paramount therapeutic goal, crucial to translating decades of molecular research into effective clinical practice.

## 1. Introduction

The discovery of nitric oxide (NO), a simple, gaseous diatomic molecule, as a pivotal signaling agent in the cardiovascular system represents a paradigm shift in modern physiology, fundamentally altering our understanding of vascular control [1,2]. For decades, the endothelium has been viewed as little more than a passive, semi-permeable barrier. This perspective was irrevocably changed by Robert F. Furchgott, who demonstrated that the vasorelaxant effect of acetylcholine was entirely dependent on an intact endothelial layer, leading him to postulate the existence of a diffusible, labile substance which was named “endothelium-derived relaxing factor” (EDRF) [3]. This discovery ignited a fervent scientific pursuit to identify this elusive molecule. The puzzle was solved through convergent work by Ferid Murad, who showed that nitrovasodilators exerted their effects via NO activation of the soluble guanylate cyclase (sGC). Finally, Louis J. Ignarro provided definitive biochemical evidence that EDRF and NO were one and the same molecule [4,5]. The collective recognition of their work with the 1998 Nobel Prize in Physiology or Medicine underscored the magnitude of this breakthrough, establishing a new class of signaling molecules known as “gasotransmitters” and revealing the endothelium as a critical, dynamic endocrine organ [6]. The subsequent identification of the NO synthase (NOS) enzyme family provided the mechanistic basis for NO production, cementing its status as a master regulator of cardiovascular homeostasis [7,8,9].

While the canonical NO-sGC-cyclic guanosine monophosphate (cGMP) pathway remains a cornerstone of vascular biology, the past three decades of research have unveiled a far more intricate and nuanced reality [10,11]. The biological impact of NO is not a simple function of its production rate but is dictated by a complex, dynamic interplay of synthesis, transport, targeted signaling, and metabolic inactivation [12,13]. To provide a coherent intellectual structure for this complexity, this review is organized around the unifying conceptual framework of “Nitric Oxide Equilibrium” (NOE). NOE is defined as the integrated, homeostatic balance between the rate of NO synthesis by NOS isoforms, the determinants of its local bioavailability, and the fidelity of its downstream signaling effects. This equilibrium is critically governed by the cellular redox environment, where NO competes with reactive oxygen species (ROS) like superoxide for its very existence. Notably, this reaction does not merely scavenge NO but generates the highly cytotoxic oxidant peroxynitrites (ONOO^−^) [14,15]. Therefore, NOE is not a static set-point but a precarious, constantly adjusting state that ensures NO signaling is precisely tailored in magnitude, location, and duration to meet physiological demands, from moment-to-moment regulation of blood pressure to long-term vascular remodeling.

From this perspective, the genesis and progression of cardiovascular disease can be re-conceptualized as a fundamental failure to maintain NOE. Pathological states such as hypertension, diabetes, and hypercholesterolemia impose a state of systemic oxidative stress that profoundly disrupts this equilibrium [16,17,18]. This disruption manifests as a dual condition: a relative or absolute deficiency of bioactive NO, leading to endothelial dysfunction, and a simultaneous excess of ONOO^−^, which drives cellular damage through protein nitration and lipid peroxidation [19,20]. This framework moves the focus beyond a simple “NO deficiency” model to a more sophisticated understanding of a systemic imbalance, where the consequences of NOE disruption include not only impaired vasodilation but also unchecked platelet aggregation, chronic inflammation through leukocyte adhesion, and maladaptive proliferation of smooth muscle cells.

The central thesis of this review is, therefore, that the dysregulation of NOE represents a foundational, common pathogenic pathway that unifies a diverse spectrum of cardiovascular diseases, making its restoration a paramount therapeutic goal [21]. In this review, we will therefore comprehensively examine the biology of NO through the lens of the NOE framework. In particular, we will begin by dissecting the foundational pillars of NOE, detailing the intricate architecture and regulation of the NOS isoforms and exploring the critical determinants of NO bioavailability, including the roles of oxidative stress, endogenous inhibitors like asymmetric dimethylarginine (ADMA), and the emerging influence of the gut microbiome’s nitrate-reducing capacity [22,23,24]. Subsequently, we will explore the multifaceted physiological roles of a maintained NOE in cardiovascular homeostasis, spanning vascular tone, cardiac function, hemostasis, and mitochondrial bioenergetics [25]. We will then systematically analyze how a disrupted NOE precipitates the pathophysiology of major cardiovascular diseases, including atherosclerosis, heart failure, and ischemia–reperfusion injury [26,27,28]. A critical evaluation of therapeutic strategies aimed at restoring NOE will follow, covering established interventions like organic nitrates and phosphodiesterase-5 (PDE5) inhibitors, as well as, next-generation approaches such as sGC modulators, targeted nanocarriers, and microbiome-based strategies [29,30,31]. Finally, by identifying critical methodological barriers in NO research and key unresolved biological questions, we will chart a course for future investigations, aiming to translate our intricate molecular understanding of NOE into a new generation of personalized and effective clinical interventions [32].

## 2. Foundations of NOE

The maintenance of NOE is contingent upon a tightly regulated and highly integrated series of biological processes. At its core, NOE is determined by the precise control of NO synthesis, the myriad factors dictating its bioavailability, and its ultimate metabolic fate. Understanding these foundational pillars is essential to appreciate how this seemingly simple molecule can exert such profound and diverse effects on cardiovascular physiology, and how subtle perturbations in these core processes can cascade into systemic pathology. This section will deconstruct the fundamental architecture of NOE, beginning with the enzymatic machinery responsible for NO production, the NOS, and then exploring the critical determinants of NO bioavailability, including the ever-present threat of oxidative stress and the newly appreciated contributions from the gut microbiome. These foundational elements collectively create the biochemical landscape upon which NOE is either sustained or disrupted.

### 2.1. Enzymatic Production: NOS Isoform Architecture and Regulation

The enzymatic synthesis of NO from its substrate, the amino acid L-arginine, is catalyzed by a family of complex, multi-domain enzymes known as NOS [33,34]. Three distinct isoforms have been identified, each encoded by a separate gene and named according to the tissue from which it was first isolated: neuronal NOS (nNOS or NOS1), inducible NOS (iNOS or NOS2), and endothelial NOS (eNOS or NOS3) [35]. While all three isoforms catalyze the same five-electron oxidation of L-arginine to produce L-citrulline and NO, they exhibit critical differences in their regulation, expression patterns, and subcellular localization, which in turn dictate their specific physiological and pathological roles [36]. Structurally, all NOS isoforms are functional homodimers and each monomer comprises two principal domains, a C-terminal reductase domain and an N-terminal oxygenase domain, linked by a calmodulin-binding peptide sequence [37]. The reductase domain is homologous to cytochrome P450 reductase and contains binding sites for nicotinamide adenine dinucleotide phosphate (NADPH), flavin adenine dinucleotide (FAD), and flavin mononucleotide. It functions to transfer electrons from NADPH to the oxygenase domain. The oxygenase domain contains a heme prosthetic group, a binding site for the essential cofactor (6R)-5,6,7,8-tetrahydrobiopterin (BH_4_), and the substrate L-arginine binding site [38]. The intricate flow of electrons from the reductase domain of one monomer to the oxygenase domain of the other is a critical feature of the dimeric structure, making dimerization an absolute prerequisite for enzymatic activity [39].

The regulation of the constitutive isoforms, eNOS and nNOS, is primarily controlled by intracellular calcium (Ca^2+^) concentrations [40]. In response to stimuli such as shear stress or agonists like acetylcholine, intracellular Ca^2+^ levels rise, promoting the binding of the Ca^2+^/calmodulin complex to the enzyme. This binding event induces a conformational change that facilitates electron flow from the reductase to the oxygenase domain, initiating the synthesis of small, transient “puffs” of NO for physiological signaling [41]. In contrast, the expression of iNOS is transcriptionally regulated and largely independent of intracellular Ca^2+^ levels, as calmodulin is so tightly bound to iNOS that it is effectively active at basal Ca^2+^ concentrations [42]. iNOS is not typically expressed in quiescent cells but is robustly induced by pro-inflammatory cytokines and microbial products (e.g., lipopolysaccharide, LPS) in immune cells like macrophages [43]. Once expressed, iNOS produces large, sustained floods of NO, often several orders of magnitude greater than that produced by the constitutive isoforms, which functions as a cytotoxic component of the innate immune response and contributes significantly to pathology in chronic inflammatory states [44].

Beyond Ca^2+^ calcium–calmodulin binding, the activity of NOS isoforms, particularly eNOS, is exquisitely fine-tuned by a complex interplay of post-translational modifications (PTMs) and subcellular localization [45]. For eNOS, localization to the caveolae of the plasma membrane through myristoylation and palmitoylation is critical for its regulation, placing it in close proximity to signaling partners and regulatory proteins like caveolin-1, which acts as a negative regulator of eNOS activity [46]. Furthermore, eNOS activity is powerfully modulated by phosphorylation at multiple sites. For instance, phosphorylation at serine 1177 (Ser1177) by the kinase protein kinase B (Akt), a downstream effector of insulin and vascular endothelial growth factor, markedly increases enzyme activity by enhancing electron flux [47]. Conversely, phosphorylation at threonine 495 (Thr495) by protein kinase C may inhibit its activity [48]. This complex code of PTMs and precise spatial organization allows for the dynamic integration of multiple signaling inputs, ensuring that eNOS-derived NO production can be tightly coupled to the physiological needs of the endothelium. Table 1 summarizes the key characteristics of the three NOS isoforms.

### 2.2. Determinants of NO Bioavailability: The Redox Balance

The enzymatic production of NO, however tightly regulated, represents only the initial step in determining its ultimate physiological impact. The true measure of NO’s functional capacity is its bioavailability, which refers to the effective concentration of bioactive NO available to reach its intended targets, such as sGC [49]. NO bioavailability is critically and perpetually challenged by the local redox environment, most notably by the presence of ROS [50]. Superoxide represents the most significant threat, reacting with NO to form ONOO^−^. It is critical to note that this reaction is diffusion-limited, proceeding at a rate constant (k ≈ 1.9 × 10^10^ M^−1^s^−1^) that is significantly faster than the dismutation of superoxide by its primary physiological scavenger, superoxide dismutase (SOD) (k ≈ 2 × 10^9^ M^−1^s^−1^) [51,52]. However, under basal conditions, the high intracellular concentration of SOD effectively outcompetes NO for superoxide. The pathological accumulation of ONOO^−^ be-comes the dominant pathway only when the production fluxes of both NO and superoxide rise simultaneously to micromolar levels, at which point the local concentration of re-actants exceeds the buffering capacity of SOD [53]. Once formed, ONOO^−^ functions as a potent and highly cytotoxic oxidant that drives cellular injury through lipid peroxidation, DNA strand breaks, and irreversible nitration of protein tyrosine residues [54]. Consequently, any pathological condition that increases superoxide production, such as inflammation, hyperglycemia, or hypercholesterolemia, inevitably disrupts NOE by simultaneously decreasing NO bioavailability and increasing peroxynitrite-mediated injury [55].

A particularly insidious mechanism that devastates NO bioavailability is the phenomenon of eNOS uncoupling [56]. Under physiological conditions, the two-step synthesis of NO is tightly coupled, with electron flow from the reductase domain to the oxygenase domain resulting in NO production. However, this process can become “uncoupled” if there is a deficiency in either the substrate L-arginine or, more critically, the essential cofactor BH_4_ [57]. In a state of BH_4_ deficiency, the electron flow within the eNOS dimer becomes dysregulated. Instead of being transferred to L-arginine, the electrons are diverted to molecular oxygen, causing the enzyme itself to produce superoxide instead of NO [58]. This pathological switch transforms eNOS from a protective, NO-producing enzyme into a source of ROS, creating a vicious cycle that further depletes NO, generates more ONOO^−^, and oxidizes any remaining BH_4_, thus locking the enzyme in a dysfunctional, uncoupled state [59].

Endogenous competitive inhibitors of NOS enzymes also play a crucial role in modulating NO bioavailability [60]. The most significant of these is ADMA, a methylated L-arginine analog produced during the routine turnover of methylated proteins [61]. ADMA competes with L-arginine for the active site of all three NOS isoforms, thereby acting as a potent endogenous inhibitor of NO synthesis [62]. In healthy individuals, ADMA is efficiently metabolized and cleared by the enzyme dimethylarginine dimethylaminohydrolase (DDAH) [63]. However, in many cardiovascular disease states, DDAH activity is suppressed by oxidative stress, leading to the systemic accumulation of ADMA [64]. Elevated plasma ADMA levels are now recognized as a strong, independent risk factor for endothelial dysfunction, hypertension, and adverse cardiovascular events, as they directly reduce the capacity for endogenous NO production [65]. Finally, NO bioavailability is also influenced by crosstalk with other endogenous gasotransmitters, namely carbon monoxide and hydrogen sulfide [66]. These molecules share several signaling pathways with NO and can modulate its production and effects, although the precise nature of these interactions in cardiovascular health and disease is complex and remains an area of active investigation [67]

### 2.3. Gut Microbiota-Derived Nitric Oxide

Beyond enzymatic synthesis of NO by NOS isoforms, a remarkable microbiome-dependent pathway for NO generation exists within the gastrointestinal tract [68]. This NOS-independent mechanism fundamentally shifts our understanding of NO homeostasis, opening new perspectives on cardiovascular health [69]. This pathway relies on the metabolic versatility of gut microbial communities, which catalyze the sequential reduction of inorganic nitrate (NO^3−^) to nitrite (NO^2−^) and ultimately to NO under specific physicochemical conditions [70]. The process begins with the consumption of dietary nitrate, a nutrient abundant in green leafy vegetables and root crops, such as beetroot [69]. After ingestion, nitrate is absorbed primarily in the proximal small intestine [70]. Within the gut lumen, nitrate serves as an alternative electron acceptor for anaerobic respiration in select bacterial taxa, a process that becomes particularly important under hypoxic conditions characteristic of the distal intestine [71].

Specific bacterial enzymes drive this transformation. Nitrate reductases catalyze the two-electron reduction of NO^3−^ to NO^2−^ [71]. Subsequently, nitrite undergoes non-enzymatic disproportionation to NO in acidic microenvironments, a conversion facilitated by reducing conditions created by dietary polyphenols and endogenous reductants, like ascorbate [72]. The NO generated through this process diffuses across the intestinal epithelium into the portal circulation, thereby contributing to systemic NO bioactivity and vascular homeostasis [73]. Importantly, this gut microbiota-driven pathway becomes critical under challenging physiological conditions. When NOS activity is compromised due to hypoxia, acidosis, or oxidative stress, this alternative mechanism provides a NOS-independent reservoir of NO [74]. In doing so, it safeguards vascular tone, platelet function, and inflammatory regulation precisely when enzymatic NO synthesis is attenuated [75]. Experimental models have demonstrated that nitrate supplementation restores NO bioavailability and improves endothelial function even when eNOS activity is impaired, underscoring the compensatory role of this pathway [76].

However, this protective system is vulnerable to disruption. Perturbations in gut microbial ecology, often driven by factors such as antibiotic exposure, high-fat diets, or chronic inflammation, deplete key nitrate-reducing taxa, including Enterobacteriaceae and Bacteroidetes [76]. This dysbiosis not only diminishes the generation of nitrite but also triggers a detrimental metabolic shift, the resulting deficiency in gut-derived NO removes a key physiological brake on vascular inflammation. Consequently, NADPH oxidase is upregulated, leading to an excessive generation of ROS within the vascular wall that accelerates the degradation of any residual nitrite and further destabilizes NOE. This combination of reduced synthesis and accelerated oxidative degradation contributes significantly to endothelial dysfunction and systemic inflammation [77]. These disruptions establish a direct mechanistic link between gut health and cardiovascular risk, positioning the microbiota as a critical determinant of NO homeostasis. Recognizing this connection opens new therapeutic possibilities: for instance, probiotic interventions targeting nitrate-reducing bacteria may represent a promising approach for enhancing gut-mediated NO production. Nevertheless, clinical validation of these strategies remains ongoing and constitutes an important frontier in cardiovascular therapeutics [78].

### 2.4. The Challenge of Quantifying NOE

Despite the clear theoretical framework of NOE, a primary and persistent challenge in translating this biology remains the inherent difficulty in direct and quantitative measurement of NO itself [79]. The molecule’s ephemeral nature and high reactivity have forced a reliance on indirect or artifact-prone methods, such as fluorescent probes with questionable specificity or assays for stable metabolites like nitrate and nitrite, which often sacrifice crucial spatial and temporal information [80]. This measurement problem is magnified when attempting to dissect the cGMP-independent roles of NO, particularly the S-nitrosoproteome [81]. Distinguishing specific, functional S-nitrosylation events from the vast background of non-specific modifications remains a formidable task, making it difficult to validate the physiological relevance of many putative NO targets [82]. Consequently, it is important to acknowledge that much of our understanding of NO signaling relies on these indirect measures, requiring a careful interpretation of experimental data in light of potential artifacts. This limitation underscores the need for more advanced, real-time biosensors to definitively map NOE dynamics in human disease.

### 2.5. Temporal Dynamics and Kinetics of NO Signaling

To fully appreciate the physiological role of NO, one must consider not just its concentration, but its temporal dynamics as well. NO is a gas with a remarkably short biological half-life, estimated to range from 0.1 to 2 s in vivo, depending on the local concentration of scavengers like hemoglobin and superoxide [83]. This fleeting existence dictates its sphere of influence; NO acts strictly as a paracrine or autocrine signaling molecule. With a diffusion coefficient of approximately 3300 µm^2^/s, a single NO molecule can traverse a radius of 100–200 µm, allowing it to cross cellular membranes and influence nearly 2 million surrounding cells within a few seconds before being metabolized [84]. This kinetic profile allows NO to act as a rapid, localized synchronizer of tissue function (e.g., coordinating the relaxation of a specific vascular bed) without causing systemic hypotension.

The signaling “message” of NO is further encoded by the temporal pattern of its synthesis, particularly for the calcium-dependent isoforms (eNOS and nNOS). These enzymes do not merely switch “on” or “off”; they respond to the complex temporal code of intracellular Ca^2+^ oscillations. Under physiological conditions, such as laminar shear stress, endothelial cells generate repetitive Ca^2+^ spikes or oscillations rather than a sustained plateau. This frequency-encoded signal triggers pulsatile bursts of NO production, which efficiently maintain vascular tone while minimizing oxidative stress [85]. In contrast, pathological stimuli often trigger a sustained, high-amplitude Ca^2+^ flood (or, in the case of iNOS, calcium-independent continuous activity). This leads to a persistently elevated NO output that saturates physiological targets such as sGC and rapidly reacts with superoxide to form ONOO^−^ [86]. Thus, the transition from physiological signaling to pathological toxicity is often defined not just by how much NO is made, but how long and in what pattern it is sustained.

## 3. Physiological Functions: Maintaining NOE in Cardiovascular Homeostasis

A properly maintained NOE is fundamental to nearly every aspect of cardiovascular health. It acts as a master regulator ensuring that the circulatory system functions seamlessly. When NOE is in physiological balance, precise, localized production of NO orchestrates a wide array of protective processes. These range from the instantaneous control of vascular tone and blood pressure to the long-term maintenance of a non-thrombogenic and anti-inflammatory endothelial surface. Beyond the vasculature, NO signaling influences cardiac performance, vascular repair mechanisms, and cellular energy metabolism through its integration with mitochondrial function.

### 3.1. Vascular Function and Homeostasis

NO is the principal regulator of vascular tone, responding to hemodynamic forces such as shear stress from blood flow. The canonical mechanism operates through the sGC–cGMP–protein kinase G (PKG) pathway, eNOS produces NO, which diffuses into vascular smooth muscle cells (VSMCs) and binds to sGC [87]. This activation converts guanosine triphosphate (GTP) to cyclic GMP, triggering PKG-mediated phosphorylation of downstream effectors that reduce intracellular Ca^2+^ and desensitize the contractile apparatus, resulting in VSMC relaxation and vasodilation. This pathway underlies flow-mediated dilation (FMD), a key measure of endothelial health. Beyond cGMP-dependent signaling, NO modulates vascular function through S-nitrosylation, the covalent attachment of an NO moiety to cysteine residues on target proteins. S-nitrosylation of large-conductance Ca^2+^-activated potassium channels increases their open probability, promoting potassium efflux, membrane hyperpolarization, and closure of voltage-gated Ca^2+^ channels, thereby contributing to vasodilation. Additionally, S-nitrosylation of nuclear factor kappa-light-chain-enhancer of activated B cells (NF-κB) within the endothelium suppresses the expression of pro-inflammatory adhesion molecules, maintaining a quiescent, anti-inflammatory phenotype [88].

Critically, the healthy endothelium maintains a non-thrombogenic surface through NO-mediated inhibition of both platelets and leukocytes. In platelets, NO activates the sGC–cGMP–PKG cascade; PKG then phosphorylates key substrates including vasodilator-stimulated phosphoprotein and inositol 1,4,5-trisphosphate receptor-associated cGMP-kinase substrate [89]. These actions prevent the intracellular Ca2+ rise and conformational changes required for platelet activation, effectively raising the aggregation threshold in response to agonists like thrombin or collagen [90]. In leukocytes, NO suppresses the master inflammatory transcription factor NF-κB, preventing its translocation to the nucleus and subsequent expression of adhesion molecules including vascular cell adhesion molecule-1 (VCAM-1) and intercellular adhesion molecule-1. By blocking these recruitment signals, NO renders the endothelial surface resistant to leukocyte adhesion and prevents their infiltration into the subendothelial space. Recent evidence further indicates that NO inhibits the nucleotide-binding domain and leucine-rich repeat pyrin domain containing 3 (NLRP3) inflammasome, suppressing the production of pro-inflammatory cytokines interleukin 1β and interleukin-18, broadening its anti-inflammatory profile [91].

### 3.2. Cardiac Electromechanical Function

NO exerts complex regulatory control over cardiac function, influencing both mechanical contraction–relaxation and electrical properties. Its effects are biphasic and depend on the NOS isoform involved, NO concentration, and the local redox environment. Under physiological conditions, low nanomolar concentrations of eNOS- and nNOS-derived NO may enhance cardiac lusitropy primarily through the cGMP–PKG pathway [92]. PKG phosphorylates phospholamban (PLB) at the sarcoplasmic reticulum, relieving inhibition of the sarcoendoplasmic reticulum calcium ATPase2 (SERCA2) and accelerating Ca^2+^ reuptake from the cytosol. Simultaneously, PKG phosphorylates cardiac troponin I (cTnI), decreasing myofilament Ca^2+^ sensitivity. The combined effect is faster Ca2+ dissociation from the contractile apparatus and improved diastolic filling [93].

In contrast, pathological states, such as sepsis or advanced heart failure, may produce micromolar concentrations of iNOS-derived NO with profoundly detrimental effects. Indeed, excessive NO directly suppresses myocardial contractility through both cGMP-dependent and cGMP-independent mechanisms [94]. High-concentration NO reacts with superoxide in the presence of oxidative stress to form ONOO^−^, which inflicts direct damage on the ryanodine receptor and SERCA2, impairing Ca^2+^ handling and severely suppressing cardiac contractility. This iNOS-driven contractile dysfunction characterizes septic cardiomyopathy and contributes to maladaptive remodeling in late-stage heart failure. NO signaling also modulates cardiac electrophysiology by influencing L-type Ca^2+^ channels and potassium channels, affecting action potential duration and arrhythmogenesis [95]. Additionally, NO can uncouple β-adrenergic signaling, blunting the heart’s response to sympathetic stimulation.

### 3.3. NO-Mediated Control of Mitochondrial Function

The regulatory influence of NO extends deep into the cell, directly integrating with the primary hub of cellular energy metabolism: the mitochondrion. Far from being a passive bystander, the mitochondrion is both a source and a target of NO signaling. This creates a complex feedback system that positions NO as a bioenergetic regulator capable of fine-tuning cellular metabolism in response to physiological cues [96].

This interaction is most profoundly demonstrated by NO’s direct, reversible inhibition of cellular respiration. NO competes with molecular oxygen for the binding site on cytochrome c oxidase, also known as Complex IV, the terminal enzyme of the electron transport chain (ETC). This binding event transiently inhibits the electron transfer to oxygen, effectively putting a “brake” on mitochondrial respiration [97]. Under physiological conditions, this is a crucial regulatory mechanism. By modulating the rate of oxygen consumption, NO can match cellular energy production to substrate availability, prevent excessive mitochondrial polarization, and importantly, divert oxygen to other cellular processes [98]. Beyond its direct effects on the ETC, NO is a significant regulator of mitochondrial biogenesis, the process of generating new mitochondria. This function is primarily mediated through the activation of peroxisome proliferator-activated receptor-gamma co-activator 1-alpha (PGC-1α), the master regulator of mitochondrial biogenesis [99]. NO, through the sGC-cGMP-PKG signaling pathway, can increase the expression and activity of PGC-1α. Activated PGC-1α then co-activates nuclear transcription factors, such as nuclear respiratory factor 1 (NRF-1) and 2 (NRF-2). These factors, in turn, drive the expression of a broad suite of genes required for mitochondrial replication and function, including mitochondrial transcription factor A [100]. The stimulation of this cascade enables physiological concentrations of NO to induce mitochondrial biogenesis and augments oxidative metabolism, which constitutes a primary adaptive mechanism to stimuli like prolonged aerobic activity [101].

Furthermore, NO signaling is intricately involved in regulating mitochondrial dynamics, the continuous cycle of fission (division) and fusion (merging). This cycle governs mitochondrial shape, size, and quality control [102]. A healthy mitochondrial network is characterized by dynamic equilibrium between these two opposing processes. Emerging evidence indicates that NO influences this balance through the S-nitrosylation of key proteins in the dynamic’s machinery [103]. Notably, NO has been shown to S-nitrosylate dynamin-related protein 1 (Drp1), a cytosolic GTPase which is considered the primary driver of mitochondrial fission. S-nitrosylation of Drp1 at a specific cysteine residue inhibits its GTPase activity and prevents its translocation to the mitochondrial outer membrane, thereby suppressing mitochondrial fission [104]. This action favors a more fused, elongated mitochondrial network, typically associated with greater metabolic efficiency and resistance to stress-induced apoptosis. Through these multifaceted interactions with respiration, biogenesis, and dynamics, NO is deeply embedded in the maintenance of mitochondrial health, acting as a critical signaling node that aligns cellular bioenergetics with the overall physiological state of the cardiovascular system [105].

### 3.4. Vascular Repair and Angiogenesis

NO is a central orchestrator of long-term vascular plasticity, critically regulating both repair of injured vessels and angiogenesis. These functions are indispensable for wound healing, embryonic development, and adaptive responses to chronic ischemia [106]. The endothelial progenitor cells (EPC), a bone marrow-derived stem cell population capable of differentiating into mature endothelial cells, is a key cellular mediator of vascular repair. eNOS-derived NO is the master regulator of the entire EPC life cycle, from initial mobilization to ultimate incorporation into damaged endothelium [107]. Ischemic or injured tissue signals trigger increased eNOS activity within the bone marrow microenvironment. This localized NO production activates matrix metalloproteinase-9 (MMP-9), which proteolytically cleaves c-Kit ligand, releasing EPC from their stromal niche and allowing circulation. Once mobilized, EPC recruitment and homing are guided by NO-dependent signaling. Ischemic tissues exhibit upregulated eNOS expression, creating a local chemoattractant NO gradient that directs circulating EPC to injury sites. Upon arrival, the cGMP-PKG pathway promotes EPC firm adhesion, survival, and proliferation by enhancing integrin surface expression and facilitating binding to the extracellular matrix. Furthermore, NO fosters differentiation of EPC into mature, functional endothelial cells, which is essential for re-endothelialization and restoration of a quiescent, anti-thrombotic vascular lining [108].

**Figure 1:**
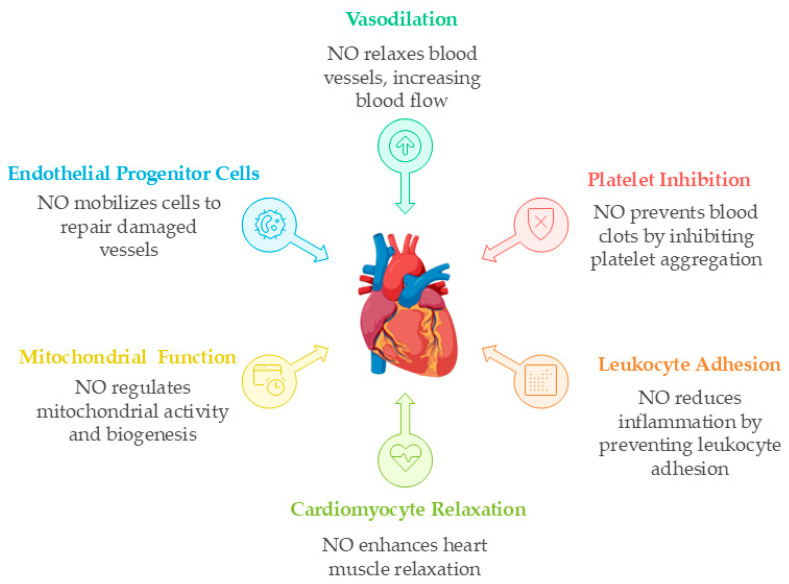
Physiological roles of NO in cardiovascular homeostasis. NO maintains vascular tone, inhibits platelet aggregation and leukocyte adhesion, enhances cardiac relaxation, promotes endothelial repair through progenitor cells mobilization and angiogenesis, and fine-tunes mitochondrial function by regulating respiration, biogenesis, and dynamics. Together, these actions preserve endothelial health and systemic cardiovascular equilibrium. NO: Nitric oxide. Created in BioRender. https://biorender.com/0100isi (accessed on 10 December 2025).

Parallel to its EPC effects, NO directly stimulates angiogenesis through crosstalk with vascular endothelial growth factor (VEGF) signaling. VEGF binding to VEGFR-2 on endothelial cells activates the phosphoinositide 3-kinase (PI3K)/Akt pathway, which phosphorylates eNOS at Ser1177, triggering NO production in a positive feedback loop. This NO not only mediates vasodilation necessary for accommodating increased blood flow but also functions as a critical downstream effector of VEGF-driven endothelial cell migration and proliferation [109]. The cGMP-PKG cascade modulates cytoskeletal dynamics required for cell motility and upregulates genes essential for cell cycle progression. By acting as both an upstream regulator of EPC mobilization and a critical downstream effector of VEGF signaling, NO is positioned as an indispensable hub in the complex network governing vascular repair and growth, highlighting its profound role in the adaptive and regenerative capacity of the cardiovascular system [110]. Figure 1 presents the multifaceted physiological roles of NO in cardiovascular homeostasis.

## 4. Dysregulation of NOE in Cardiovascular Pathologies

The elegant homeostatic balance of NOE, so vital for cardiovascular health, is profoundly vulnerable to disruption by the metabolic and inflammatory insults that characterize modern cardiometabolic diseases. When this equilibrium is shattered, NO signaling shifts from a protective, adaptive force to a maladaptive or even pathogenic one. The disruption of NOE is not merely a biomarker of disease but a fundamental, causative driver of pathology, marking the critical tipping point where physiological stress turns into cellular and tissue injury. This section will systematically explore how the failure to maintain NOE may serve as a common mechanistic thread weaving through a diverse spectrum of cardiovascular pathologies. We will begin with endothelial dysfunction, the initial and universal manifestation of NOE collapse, and then proceed to analyze its central role in the development of atherosclerosis, the maladaptive remodeling in heart failure, the temporal duality of ischemia–reperfusion injury, and the complexities of cerebrovascular disease.

### 4.1. Endothelial Dysfunction: The Common Denominator of Cardiovascular Pathologies

Endothelial dysfunction is the earliest detectable stage in the continuum of vascular disease and can be functionally defined as the comprehensive failure of NOE [111]. This state is characterized not only by impaired endothelium-dependent vasodilation but also by a profound phenotypic switch of the endothelium from a quiescent, anti-atherogenic monolayer to a pro-inflammatory, pro-thrombotic, and pro-proliferative surface [112]. This pathological transition involves increased endothelial permeability to lipoproteins, enhanced expression of leukocyte adhesion molecules, and a reduced capacity to inhibit platelet aggregation, collectively creating a permissive environment for atherogenesis. It is the universal consequence of chronic exposure to cardiovascular risk factors and serves as the common pathogenic denominator for a host of diseases, most notably hypertension and diabetes [113].

In essential hypertension, the disruption of NOE is centrally driven by the overactivity of the renin–angiotensin system. Its principal effector, angiotensin II (Ang II), acting via its type 1 receptor, orchestrates a multi-pronged assault on NO bioavailability [114]. Ang II is a powerful activator of membrane-bound NADPH oxidase enzymes, which become a primary source of superoxide. Mechanistically, Ang II signaling triggers the phosphorylation and translocation of cytosolic subunits (such as p47phox) to the membrane, leading to the assembly of the active oxidase complex (specifically NADPH Oxidase 1 and NADPH Oxidase 2), which transfers electrons from NADPH to molecular oxygen [104].

This surge in superoxide directly scavenges NO in a diffusion-limited reaction, crippling its vasodilatory capacity, while simultaneously generating the highly damaging oxidant ONOO^−^ [115]. This process alone is sufficient to induce dysfunction, but Ang II further dismantles NOE by directly targeting eNOS enzyme itself. It promotes eNOS uncoupling by downregulating the expression of GTP cyclohydrolase I, the rate-limiting enzyme in the synthesis of the critical cofactor BH4 [116]. Concurrently, Ang II signaling activates PKC, which phosphorylates eNOS at its inhibitory threonine 495 site [117]. This combination of cofactor depletion, direct enzymatic inhibition, and massive oxidative stress, transforms eNOS from a protective enzyme into a source of additional superoxide, locking the endothelium in a dysfunctional, vasoconstricted state.

**Figure 2:**
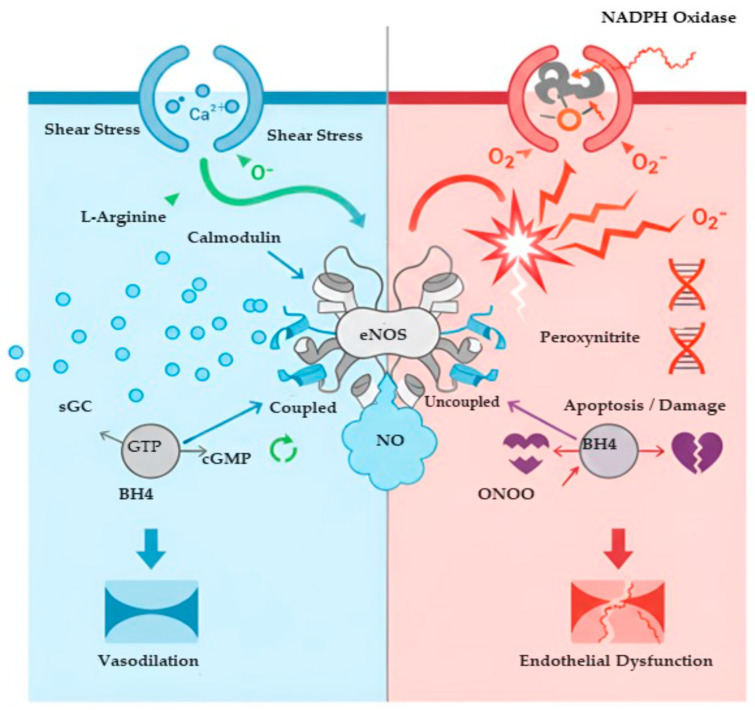
Molecular mechanisms governing the shift from Nitric Oxide Equilibrium (NOE) to endothelial dysfunction. The schematic illustrates the central role of the endothelial cell in maintaining or disrupting NOE. (Left Panel) Under physiological conditions (e.g., shear stress), intracellular calcium influx and adequate levels of the cofactor BH_4_ facilitate the dimerization of coupled eNOS. This enzyme efficiently oxidizes L-arginine to produce bioactive Nitric Oxide, which activates soluble guanylate cyclase to convert GTP to cGMP, ultimately resulting in vasodilation. (Right Panel) In pathological states, oxidative stress (e.g., from NADPH oxidase) leads to the oxidation of BH_4_. This causes eNOS uncoupling, where the enzyme switches to producing superoxide instead of NO. The rapid reaction between remaining NO and superoxide forms the highly toxic ONOO^−^, driving DNA damage, apoptosis, and endothelial dysfunction. BH_4_: tetrahydrobiopterin; Ca^2+^: calcium ion; cGMP: cyclic guanosine monophosphate; eNOS: endothelial nitric oxide synthase; GTP: guanosine triphosphate; NO: nitric oxide; O_2_^−^: superoxide anion; ONOO^−^: peroxynitrites; sGC: soluble guanylate cyclase. Created in BioRender. https://BioRender.com/td3ok7w. (accessed on 29 December 2025).

In diabetes mellitus, endothelial dysfunction arises from the complex interplay of insulin resistance, hyperglycemia, and dyslipidemia [118]. In healthy endothelium, insulin binding triggers the PI3K/Akt signaling cascade, leading to the activating phosphorylation of eNOS at Ser1177 and subsequent NO production, vital for metabolic homeostasis [119]. In a state of insulin resistance, this specific pathway is selectively impaired. The alternative, pro-atherogenic mitogen-activated protein kinase pathway of insulin signaling remains intact, leading to the overproduction of the potent vasoconstrictor endothelin-1, while NO synthesis falters [120]. This imbalance is profoundly amplified by chronic hyperglycemia, which fuels oxidative stress through multiple avenues, including the formation of advanced glycation end-products (AGEs). AGEs, by binding to their receptor on endothelial cells, trigger further NADPH oxidase activation [121]. Simultaneously, intracellular glucose overload overwhelms the mitochondrial ETC, forcing electrons to back up at Complex III and leak directly to molecular oxygen; this mitochondrial superoxide generation activates further cytosolic oxidative pathways in a vicious feed-forward loop [17]. The resulting oxidative stress environment not only consumes NO but also inhibits the enzyme DDAH, leading to the accumulation of the endogenous NOS inhibitor, ADMA [122]. This convergence of impaired insulin signaling, relentless oxidative stress, and elevated ADMA levels ensures the complete and sustained collapse of NOE, dramatically accelerating the progression of diabetic vascular complications. The molecular mechanisms governing this critical shift from physiological NO signaling to pathological oxidative stress within the endothelial cell are illustrated in Figure 2.

### 4.2. Atherosclerosis: Inflammatory Remodeling of NOE

Atherosclerosis, the underlying cause of most heart attacks and strokes, is a chronic inflammatory disease of the arterial wall that can be viewed as the ultimate pathological manifestation of a sustained failure of NOE [123]. The entire process, from the initial fatty streak to the complex, rupture-prone plaque, is orchestrated and accelerated by the progressive dysregulation of NO signaling. In a healthy vessel, the constitutive production of NO by eNOS serves as the primary anti-atherogenic defense mechanism. However, once NOE is compromised by systemic risk factors, this protective shield fails, initiating a self-perpetuating cycle of lipid accumulation, inflammation, and cellular proliferation.

The initiation of atherosclerosis begins with the failure of NO to protect the endothelial barrier. Reduced NO bioavailability increases the permeability of the endothelial monolayer, allowing low-density lipoprotein (LDL) particles to infiltrate the subendothelial space [124]. Thereafter, low-NO environment promotes the oxidative modification of LDL (oxLDL), a key pro-inflammatory trigger [125]. Concurrently, the loss of NO’s inhibitory effect on NF-κB signaling allows endothelial cells to upregulate the expression of adhesion molecules, particularly VCAM-1 [126]. This transforms the normally “slippery” endothelium into a sticky surface that captures circulating monocytes. These monocytes then transmigrate into the intima, where they differentiate into macrophages and begin to avidly engulf the accumulated oxLDL, becoming the lipid-laden “foam cells”, the pathological hallmark of the early atherosclerotic lesion.

As the lesion progresses, the role of NO signaling becomes more complex and pernicious, highlighting a critical duality between the different NOS isoforms. While the protective, eNOS-derived NO signaling remains impaired, the inflammatory microenvironment within the developing plaque induces the high-level expression of iNOS in macrophages and vascular smooth muscle cells [127]. In this confined space, already rife with superoxide from activated macrophages and dysfunctional mitochondria, the massive, sustained flux of NO from iNOS does not confer protection. Instead, it reacts almost instantaneously with superoxide to generate vast quantities of ONOO^−^ [128]. This ONOO^−^ storm dramatically accelerates disease progression by promoting further lipid peroxidation, inducing apoptosis in VSMCs (thereby destabilizing the plaque’s fibrous cap), and driving the nitration and dysfunction of critical proteins [129]. This creates a clear distinction: the loss of constitutive, low-level eNOS signaling initiates the disease, while the gain of inducible, high-level iNOS signaling within the plaque actively promotes its inflammatory progression and instability. This isoform-specific functional switch is a central feature of the pathological remodeling of NOE in atherosclerosis.

Crucially, the inflammatory landscape of the plaque is further destabilized by the recruitment of neutrophils, which act as potent effectors of oxidative and nitrosative stress. Upon activation, neutrophils undergo a “respiratory burst,” generating massive quantities of superoxide via NADPH oxidase while simultaneously releasing myeloperoxidase (MPO) from their azurophilic granules. MPO is a critical enzyme in NOE dysregulation; it catalytically consumes NO to generate nitrogen dioxide radicals (•NO_2_), thereby reducing NO bioavailability while promoting the nitration of lipoproteins and extracellular matrix proteins [130]. Furthermore, activated neutrophils release Neutrophil Extracellular Traps (NETs) decorated with MPO and histones, which provide a physical scaffold that concentrates these oxidative reactions near the endothelial surface, exacerbating dysfunction and promoting plaque rupture.

### 4.3. Heart Failure: From Adaptation to Maladaptation

In the context of heart failure (HF), a complex clinical syndrome of impaired cardiac function, the role of NO signaling is remarkably dynamic, transitioning from a crucial adaptive mechanism in the early stages of cardiac stress to a significant driver of pathology in advanced disease [131]. This evolution reflects a profound maladaptive remodeling of NOE within the myocardium itself. In the early phases, often characterized by diastolic dysfunction or HF with preserved ejection fraction (HFpEF), increased wall stress stimulates constitutive NOS isoforms to produce NO, which serves as protective and compensatory molecule. Indeed, as above reported, the eNOS-derived NO enhances lusitropy, or diastolic relaxation, by activating the PKG pathway, which phosphorylates PLB and cTnI to accelerate Ca^2+^ reuptake and myofilament dissociation [132]. This improves diastolic filling in the face of pressure overload. Concurrently, NO stimulates mitochondrial biogenesis through PGC-1α, helping to meet the increased energetic demands of the stressed myocardium [133]. In this context, NO signaling is a vital adaptive response aimed at preserving cardiac efficiency.

However, as the HF syndrome progresses, particularly towards heart failure with reduced ejection fraction (HFrEF), a pathological shift occurs. The sustained neurohormonal activation (e.g., Ang II, catecholamines) and chronic systemic inflammation that characterize advanced HF may lead to the robust induction of iNOS within cardiomyocytes [134]. This isoform, once expressed, generates a high-output, sustained flux of NO that is profoundly cardiotoxic. In the failing heart’s high-oxidative-stress environment, this iNOS-derived NO rapidly forms ONOO^−^, which directly suppresses myocardial contractility by damaging critical Ca^2+^-handling proteins, like the ryanodine receptor and SERCA2 [135]. This contributes directly to the decline in systolic function. Furthermore, this maladaptive iNOS activity promotes myocyte apoptosis and adverse extracellular matrix remodeling, further driving the progression of ventricular dysfunction. The situation is compounded by the pathological mislocalization of nNOS as well. In a healthy heart, nNOS is precisely localized in the sarcoplasmic reticulum, where it fine-tunes Ca2+ release. In the failing heart, nNOS becomes displaced to the sarcolemma, leading to dysregulated Ca^2+^ cycling, impaired excitation-contraction coupling, and an increased propensity for fatal arrhythmias [136]. This combination of maladaptive iNOS induction and nNOS mislocalization represents a complete and detrimental collapse of myocardial NOE, transforming NO from a supportive signal into a key executioner of cardiac decompensation.

### 4.4. Ischemia–Reperfusion (I/R) Injury: The Temporal Duality

I/R injury, the paradoxical exacerbation of cellular dysfunction and death that occurs upon the restoration of blood flow to previously ischemic tissue, provides a striking example of the dual nature of NO signaling [137]. The role of NO in this context is exquisitely dependent on timing, concentration, and the specific NOS isoform involved, embodying a “temporal duality” where it can be either profoundly protective or catastrophically destructive. The protective role of NO is most evident in the phenomenon of ischemic preconditioning, where brief, non-lethal episodes of ischemia protect the heart from a subsequent prolonged ischemic insult. This protection is largely mediated by eNOS-derived NO. The small bursts of NO produced during preconditioning trigger the S-nitrosylation of key mitochondrial proteins, most notably subunits of Complex I of the ETC [138]. This reversible modification partially inhibits Complex I, which paradoxically preserves mitochondrial function by reducing electron leakage and ROS production during the subsequent ischemic period. This action prevents the opening of the mitochondrial permeability transition pore upon reperfusion, a critical event that triggers mitochondrial swelling and initiates the apoptotic cell death cascade.

In stark contrast, the reperfusion phase itself is characterized by a massive and destructive burst of NO production that is central to the pathogenesis of I/R injury. The reintroduction of molecular oxygen to the ischemic tissue, which is already in a state of metabolic distress, Ca^2+^ overload and acidosis, triggers a massive surge of superoxide production from sources like NADPH oxidase and uncoupled mitochondria [139]. This oxidative assault is significantly amplified by the rapid infiltration of neutrophils, the immune system’s ‘first responders’ to reperfusion. In a phenomenon often described as the ‘lethal reperfusion injury’, activated neutrophils adhere to the post-ischemic endothelium and actively generate both high-flux NO (via iNOS) and superoxide (via NADPH oxidase) in close proximity [140]. This simultaneous production creates a localized ‘reaction chamber’ for the instantaneous formation of ONOO^−^, which overwhelms endogenous antioxidant defenses and drives necrotic cell death in the myocardium. Critically, the inflammatory environment created by the ischemic insult can also lead to the rapid transcriptional induction of iNOS [141]. The near-simultaneous convergence of a high-flux of NO from iNOS and a massive burst of superoxide from re-oxygenation creates the perfect storm for the formation of ONOO^−^. This explosive production of ONOO^−^ during the first few minutes of reperfusion is a primary executioner of cardiomyocytes death. It inflicts widespread, irreversible damage through lipid peroxidation of cellular membranes, DNA damage, and the nitration of critical enzymes and structural proteins, leading to both necrotic and apoptotic cell death [142]. Thus, in I/R injury, the carefully regulated, eNOS-driven signaling that protects mitochondria before the insult is completely overwhelmed by the chaotic, iNOS-driven production of ONOO^−^ during reperfusion, perfectly illustrating the critical importance of maintaining NOE for cell survival.

### 4.5. Stroke and Cerebrovascular Disease

In the cerebro-vasculature, maintaining NOE is paramount for the precise regulation of cerebral blood flow to meet the brain’s exceptionally high and dynamic metabolic demands [143]. This process, known as neurovascular coupling, ensures that increases in neuronal activity are met with a corresponding increase in local blood flow, a response critically dependent on NO derived from both eNOS in the endothelium and nNOS in neurons. In the event of an ischemic stroke, the abrupt cessation of blood flow triggers a profound disruption of NOE, leading to a complex and evolving pathophysiology where the different NOS isoforms play distinct and often opposing roles [144]. The initial ischemic core is surrounded by a region of moderately reduced blood flow known as the penumbra, where tissue is viable but at high risk. The fate of this penumbral tissue is largely determined by the balance of the detrimental versus protective effects of NO signaling.

The most immediate pathological role of NO in ischemic stroke is driven by nNOS and is linked to the phenomenon of excitotoxicity. The ischemic cascade leads to massive synaptic release of the excitatory neurotransmitter glutamate. This glutamate over-activates N-methyl-D-aspartate (NMDA) receptors on postsynaptic neurons, causing excessive Ca^2+^ influx. This intracellular Ca^2+^ overload potently activates nNOS, which is often physically coupled to the NMDA receptor complex [145]. The resulting burst of NO coincides with a massive surge of superoxide generated by stalled mitochondrial electron transport and xanthine oxidase activation. Consequently, the local concentrations of both radicals rise sufficiently to outcompete antioxidant defenses (such as SOD), driving the rapid, diffusion-limited reaction that yields ONOO^−^. This ONOO^−^ inflicts direct neuronal damage by causing DNA strand breaks, which activates the DNA repair enzyme poly (ADP-ribose) polymerase in a futile and energy-depleting cycle, ultimately leading to neuronal death [146].

Following the initial excitotoxic phase, a secondary wave of injury unfolds over hours to days, driven by inflammation and the induction of iNOS. Microglia, the brain’s resident immune cells, become activated in response to the ischemic damage and, along with infiltrating neutrophils and macrophages, begin to express high levels of iNOS. The sustained, high-output production of NO from iNOS generates micromolar concentrations of NO that readily react with respiratory burst-derived superoxide from phagocytes, ensuring a continuous supply of ONOO^−^. The sustained, high-output production of NO from iNOS in the post-ischemic brain would significantly contribute to secondary brain damage. It amplifies the inflammatory response, promotes apoptosis in neurons and glial cells within the penumbra, and critically, compromises the integrity of the blood–brain barrier (BBB) [147]. Peroxynitrite generated from the iNOS-derived NO damages tight junction proteins that seal the BBB, leading to increased permeability, vasogenic edema, and the potential for hemorrhagic transformation, all of which worsen clinical outcomes. In this complex landscape, the protective capacity of eNOS to promote collateral blood flow and salvage penumbral tissue is often overwhelmed by the destructive, isoform-specific actions of nNOS-driven excitotoxicity and iNOS-driven neuroinflammation, showcasing the most intricate and regionally specific disruption of NOE in cardiovascular pathology [148]. A summary of how NOE is disrupted across these major cardiovascular pathologies is presented in Table 2.

## 5. Therapeutic Strategies Targeting NOE

Since a disrupted NOE accelerates the onset and progression of cardiovascular disease, restoring this equilibrium has become a central therapeutic objective. Although traditional pharmacological agents have long targeted NO signaling, recent advances in understanding the complexities of NOE, particularly the interplay between oxidative stress and enzymatic dysfunction, have resulted in the development of more precise and sophisticated treatment strategies.

### 5.1. Pharmacological and Endogenous NO Enhancement

Therapeutic strategies aimed at restoring NO bioavailability operate across multiple levels of the signaling pathway. Direct pharmacological augmentation of NO bioavailability traditionally relies on organic nitrates such as nitroglycerin, which act as NO donors following biotransformation by mitochondrial aldehyde dehydrogenase-2 (ALDH2) [149]. However, their clinical utility is constrained by tolerance development, primarily driven by ALDH2 inactivation and systemic oxidative stress that simultaneously depletes NO. Prolonged nitroglycerin administration increases mitochondrial superoxide production, which in turn inactivates ALDH2 and scavenges NO, thereby creating a self-perpetuating cycle of enzymatic dysfunction and oxidative damage [150]. Genetic factors further influence this response; individuals carrying the ALDH22 polymorphism exhibit markedly greater tolerance, with a 67.9% reduction in nitroglycerin-mediated dilation compared to 39.9% in ALDH21 carriers [151]. While these findings suggest that genetic stratification could improve therapeutic precision, the inherent limitations of nitrate bio-activation have prompted the search for alternative donors.

Consequently, significant research interest has shifted toward the engineering of Dinitrosyl Iron Complexes (DNICs) as a novel class of NO donors. Unlike nitroglycerin, DNICs represent a physiological form of NO storage and transport that naturally occur in cells bound to glutathione or proteins. These complexes act as “working models” of the active sites of nitrosylated metalloproteins, and they are capable of delivering NO (and the nitrosonium ion, NO^+^) to specific targets without inducing the oxidative tolerance associated with the bio-activation of nitrates [152]. Recent advances in the chemical engineering of DNICs have focused on modifying the ligand environment by incorporating specific thiol or non-thiol ligands to precisely tune the kinetics of NO release and enhance water solubility. This strategic ligand engineering allows for the creation of stable, long-acting NO donors that can lower blood pressure and protect against ischemia–reperfusion injury with greater efficacy and fewer side effects than traditional donors, marking a rapidly developing frontier in NO-based pharmacotherapy [153,154].

PDE5 inhibitors, such as sildenafil, enhance endogenous NO signaling by preventing cGMP degradation, thereby prolonging the NO vasodilatory effect. This mechanism is particularly beneficial in conditions, such as pulmonary hypertension and erectile dysfunction, where maintaining elevated cGMP levels extends NO-sGC signaling without requiring additional NO synthesis [155]. Beyond the PDE5 inhibition, the sGC modulation may represent a significant therapeutic advance, too. sGC stimulators, exemplified by riociguat, increase enzyme sensitivity to residual NO through cooperative interaction with the heme moiety, enabling effective activation even at low NO concentrations. In contrast, sGC activators, such as cinaciguat, bypass NO dependence entirely by activating oxidized, heme-free sGC, a state frequently encountered under severe oxidative stress [156]. This mechanism allows cGMP generation even when NO is nearly absent, offering a therapeutic option for advanced disease characterized by profound endothelial dysfunction [157].

Strategies to enhance endogenous NO synthesis focus on substrate and cofactor availability or removal of inhibitory factors. L-arginine supplementation has demonstrated limited efficacy due to the “arginine paradox”, wherein intracellular L-arginine concentrations remain sufficient for maximal NOS activity despite increased uptake. This phenomenon likely reflects compartmentalization of arginine pools and competitive arginase activity rather than global substrate depletion [158]. L-citrulline supplementation provides a more reliable approach by bypassing hepatic first-pass metabolism and regenerating L-arginine via the argininosuccinate cycle, thereby sustaining substrate availability for eNOS [159]. Correction of eNOS uncoupling through BH4 supplementation has shown to be beneficial in genetically determined BH4 deficiency; however, its effectiveness is compromised under oxidative stress, which depletes BH4 through peroxynitrite-mediated oxidation [160]. This depletion shifts eNOS activity from NO synthesis to superoxide generation, while additional modifications, such as S-glutathionylation at critical cysteine residues, further reinforce uncoupling. Effective restoration, therefore, requires simultaneous correction of BH4 levels and normalization of the glutathione redox pool [161].

An alternative NOS-independent route for NO generation involves the dietary nitrate–nitrite–NO pathway, which becomes particularly relevant under hypoxic conditions [162]. Nitrate-rich foods, such as beetroot juice, improve endothelial function and reduce blood pressure through sequential bacterial and enzymatic reduction in saliva and tissues. Despite promising results, clinical efficacy remains variable, suggesting that differences in nitrite reductase activity and nitrite bioavailability may influence individual responses [163]. Additional research has focused on reducing endogenous NOS inhibitors like ADMA [164]. Activation of DDAH, the enzyme responsible for ADMA degradation, has also shown potential in preclinical models, although translation to clinical practice remains limited [165].

### 5.2. Advanced Delivery, Targeting, and Personalized Approaches

Advanced delivery systems aim to maximize therapeutic efficacy by confining NO-based interventions to diseased sites, thereby reducing systemic effects and tolerance. Nanoparticle platforms, including liposomes, silica-based carriers, and polymeric systems, have been engineered to release NO donors in response to specific triggers such as pH, redox status, or enzymatic activity [166]. Functionalization with targeting ligands enables selective accumulation at atherosclerotic plaques, thrombi, or inflamed endothelium. Encapsulation within liposomes has been shown to improve NO delivery by up to seven-fold compared with unencapsulated formulations, while maintaining carrier stability even in the presence of hemoglobin. Cationic liposomes further enhance vascular targeting through electrostatic interactions with endothelial cells, promoting localized accumulation [167].

Mitochondria-targeted therapies provide subcellular precision, offering protection against oxidative injury during ischemia or HF. Compounds such as MitoQ, a ubiquinone derivative conjugated to a triphenylphosphonium moiety, selectively accumulate within the mitochondrial matrix. This localization enables scavenging of superoxide, restoration of membrane potential, and improvement of oxidative phosphorylation in pressure-overload HF. These effects collectively reduce hydrogen peroxide formation, enhance cardiac contractility, and preserve Ca^2+^ retention capacity [168]. In addition, inhaled NO represents a clinically validated approach for obtaining selective pulmonary vasodilation in conditions such as acute respiratory distress syndrome and pulmonary hypertension. Rapid inactivation by hemoglobin confines its action to ventilated lung regions, improving ventilation–perfusion matching and oxygenation without systemic hypotension [169]. This selectivity permits the use of higher local concentrations while avoiding adverse hemodynamic effects.

Effective restoration of NO equilibrium increasingly relies on personalized strategies informed by robust biomarkers and genetic profiling [170]. Functional assessments, such as FMD of the brachial artery, provide real-time evaluation of integrated NO bioavailability, reflecting the vasodilatory response to shear stress-induced eNOS activation. Impaired FMD correlates with atherosclerotic burden, aneurysm progression, and future cardiovascular risk, establishing its role as a prognostic marker [171]. Biochemical indicators, including plasma ADMA, nitrate/nitrite levels, and nitrosothiols, also offer complementary insights into individual pathophysiology. Plasma nitrite, a surrogate for basal NOS activity, is reduced by up to 70% in endothelial dysfunction, and longitudinal monitoring can sensitively track therapeutic response [172]. Nitrosothiol formation further reflects residual NO bioavailability and successful S-nitrosation of regulatory proteins [173]. Patient-specific variables such as sex, age, and genetic polymorphisms significantly influence susceptibility and treatment outcomes. Variants in the *eNOS* gene, including T-786C and G894T, impair transcription and enzymatic activity, respectively, and are associated with altered responsiveness to statins and NO-augmenting therapies [174]. Sex-related differences in estrogen-dependent eNOS expression and oxidative stress patterns further define therapeutic windows [175]. Integrating these factors into clinical decision-making enables rational, individualized therapy design that addresses the underlying defect in NO homeostasis.

### 5.3. Clinical Trial Insights and Future Directions

Negative outcomes from NO-pathway trials underscore the limitations of overly simplistic therapeutic strategies. The VINTAGE MI trial, which evaluated L-arginine supplementation following myocardial infarction, was terminated prematurely due to increased mortality in the treatment group, with six deaths (8.6%) compared to none in the placebo arm (*p* = 0.01). This result demonstrates that excess substrate does not correct rate-limiting defects, such as eNOS uncoupling, cofactor depletion, or oxidative damage [176]. In advanced disease characterized by high oxidative stress, additional L-arginine may exacerbate pathology by fueling superoxide generation from uncoupled eNOS rather than promoting NO synthesis, thereby intensifying oxidative injury and ischemia [177].

Mechanistically, the failure of VINTAGE MI reflects a fundamental principle: when eNOS is uncoupled and mitochondrial function is impaired, exogenous substrate cannot restore enzymatic coupling and instead drives aberrant ROS production [178]. The elderly subgroup (≥60 years) exhibited disproportionate mortality (15.5%, *p* = 0.02 versus placebo), suggesting that age-related oxidative stress amplifies this risk. These findings have redirected research towards strategies that stabilize cofactors, recouple eNOS, and incorporate biomarker-guided patient selection [179]. This shift validates a mechanism-driven, precision approach over universal supplementation and highlights the necessity of tailoring interventions to individual pathophysiology for effective restoration of NOE.

## 6. Critical Challenges and Future Directions

While NO is indisputably a central molecule in cardiovascular biology, the translation of this knowledge into revolutionary clinical practice is constrained by a deeply interconnected web of biological and clinical challenges [180]. The methodological limitations surrounding NO measurement, as outlined in Section 2.4, have created significant gaps in our fundamental biological understanding. For instance, while we know eNOS and nNOS are both constitutively expressed in the heart, their distinct, non-redundant roles in long-term pathological remodeling remain obscure precisely because we lack the tools to track their isoform-specific NO production and downstream targets in vivo [181]. Similarly, we lack a quantitative definition of the “redox threshold” at which oxidative stress causes an irreversible collapse of NOE [182].

Identifying a measurable ratio of superoxide to NO that marks this “point of no return” would be a monumental step towards staging vascular disease, but it is a goal that remains difficult without more advanced, real-time biosensors [183]. These knowledge gaps create significant hurdles for clinical implementation and contribute to the failure of promising therapeutic concepts in large-scale trials. The disappointing outcomes of many L-arginine trials, for example, can be attributed to a failure to appreciate these complexities; they were conducted without biomarkers capable of selecting patients who were actually “substrate-deficient” and were applied in advanced disease states where the entire NOE system, from eNOS coupling to sGC responsiveness, was already profoundly compromised [184].

Therefore, the field’s future hinges on an integrated, multi-pronged approach. The development of novel, specific, and real-time NO sensors is a critical priority that will unlock answers to many of our most pressing biological questions. This must be paired with a shift towards more sophisticated, biomarker-guided clinical trial designs. Future trials for NO-based therapies should move beyond a “one-size-fits-all” model and use biomarkers like ADMA and FMD, or even genetic polymorphisms in the eNOS gene, to stratify patients and enroll those most likely to respond to a specific intervention [185]. Finally, there is a pressing need to address remaining gaps in clinician education and to develop standardized, validated assays that can be reliably implemented in a clinical setting. Only by tackling these methodological, biological, and clinical challenges in concert can we hope to translate our intricate molecular understanding of NOE into a new generation of personalized and effective cardiovascular medicine.

## 7. Conclusions

NO, despite its molecular simplicity, plays a multifaceted and indispensable role in cardiovascular physiology [186]. This review has examined NOE as a central regulatory axis, integrating the synthesis, bioavailability, and signaling dynamics of NO into a cohesive framework for understanding vascular health [187]. The maintenance of NOE is essential for endothelial function, anti-inflammatory signaling, and metabolic homeostasis [188]. Conversely, its disruption, whether through oxidative stress, enzymatic uncoupling, or endogenous inhibition emerges as a convergent mechanism underlying endothelial dysfunction, atherogenesis, and maladaptive cardiac remodeling [189].

Therapeutic strategies aimed at restoring NOE must move beyond generalized NO supplementation or substrate repletion. The complexity of NO biology demands precision-based approaches capable of identifying and correcting specific modes of dysregulation. Emerging technologies, including biomarker-guided phenotyping and targeted delivery systems, offer promising avenues for restoring NO balance in a spatially and temporally controlled manner [190]. These innovations represent a paradigm shift from reactive treatment of established disease to proactive preservation of vascular integrity. In summary, restoring and maintaining NOE should be recognized not merely as a therapeutic goal, but as a foundational principle in the prevention and management of cardiovascular disease. Future research must continue to refine our understanding of NOE and translate this knowledge into clinically actionable strategies that uphold vascular health across the lifespan.

## Figures and Tables

**Table 1:** Characteristics and Regulation of NOS Isoforms.

Feature	nNOS (NOS1)	iNOS (NOS2)	eNOS (NOS3)	References
Primary Regulation	Controlled by intracellular Ca^2+^ concentrations. Ca^2+^/calmodulin binding is required for activity.	Transcriptionally regulated by pro-inflammatory cytokines and microbial products (e.g., LPS). Largely independent of Ca^2+^ as calmodulin is tightly bound.	Controlled by intracellular Ca^2+^ concentrations. Also finely tuned by PTMs and subcellular localization.	[40,41,42,43,44,45]
Expression & Localization	First isolated from neuronal tissue.	Not typically in quiescent cells; robustly induced in immune cells like macrophages.	First isolated from endothelial tissue. Localized to caveolae of the plasma membrane via myristoylation and palmitoylation.	[35,43,46]
NO Production	Produces small, transient “puffs” of NO for physiological signaling.	Produces large, sustained floods of NO, often several orders of magnitude greater than constitutive isoforms.	Produces small, transient “puffs” of NO for physiological signaling in response to stimuli like shear stress.	[41,44]
Primary Function	Physiological signaling.	Cytotoxic component of the innate immune response.	Physiological signaling (e.g., vasodilation in response to shear stress or agonists).	[41,44]
Pathological Role	Can contribute to pathologies when dysregulated (e.g., excitotoxicity in stroke).	Can contribute significantly to pathology in chronic inflammatory states.	Dysregulation (e.g., uncoupling) is a key driver of cardiovascular pathologies like endothelial dysfunction and atherosclerosis.	[44]
Key Specific Features	Constitutive isoform.	Inducible isoform.	Constitutive isoform. Activity is powerfully modulated by phosphorylation (e.g., activating at Ser1177 by Akt, inhibitory at Thr495 by PKC) and interaction with caveolin-1.	[47,48]

Akt: protein kinase B; Ca^2+^: calcium ion; eNOS (NOS3): endothelial nitric oxide synthase; iNOS (NOS2): inducible nitric oxide synthase; LPS: lipopolysaccharide; NO: nitric oxide; nNOS (NOS1): neuronal nitric oxide synthase; PKC: protein kinase C; PTMs: post-translational modifications; Ser1177: serine residue at position 1177 (phosphorylation site on eNOS); Thr495: threonine residue at position 495 (phosphorylation site on eNOS).

**Table 2:** Summary of NOE Disruption in Cardiovascular Pathologies.

Pathology	Primary Drivers of NOE Disruption	Key NOS Isoform(s) Involved	Primary Pathological Consequence	References
Endothelial Dysfunction	Oxidative stress (e.g., from Ang II, hyperglycemia), ADMA accumulation, BH_4_ deficiency	eNOS (impaired activity and uncoupling)	Impaired vasodilation, pro-inflammatory and pro-thrombotic endothelial phenotype	[111,112,113,114,115,116,117,118,119,120,121,122]
Atherosclerosis	LDL oxidation, chronic inflammation, superoxide production from macrophages	eNOS (loss of protective function), iNOS (pro-inflammatory signaling within plaque)	Monocyte adhesion, foam cell formation, plaque progression and instability	[123,124,125,126,127,128,129,130]
Heart Failure	Neurohormonal activation (Ang II, catecholamines), chronic inflammation, oxidative stress	eNOS (adaptive early), iNOS (maladaptive induction), nNOS (maladaptive mislocalization)	Contractile suppression (systolic dysfunction), impaired relaxation (diastolic dysfunction), arrhythmogenesis	[131,132,133,134,135,136]
Ischemia–Reperfusion Injury	Reoxygenation-induced superoxide burst, inflammation, calcium overload	eNOS (protective in preconditioning), iNOS (induced during reperfusion)	Massive peroxynitrites formation leading to cardiomyocyte apoptosis and necrosis	[137,138,139,140,141,142]
Stroke & Cerebrovascular Disease	Excitotoxicity (glutamate), neuroinflammation, BBB disruption	nNOS (excitotoxicity), iNOS (neuroinflammation, BBB damage), eNOS (impaired collateral flow)	Neuronal death, vasogenic edema, secondary brain injury	[143,144,145,146,147,148]

ADMA: asymmetric dimethylarginine; Ang II: angiotensin II; BBB: blood–brain barrier; BH_4_: tetrahydrobiopterin; eNOS (NOS3): endothelial nitric oxide synthase; iNOS (NOS2): inducible nitric oxide synthase; LDL: low-density lipoprotein; nNOS (NOS1): neuronal nitric oxide synthase; NOE: nitric oxide equilibrium.

## Data Availability

The original contributions presented in this study are included in the article. Further inquiries can be directed to the corresponding author.
